# Chemical Contamination of Green Turtle (*Chelonia mydas*) Eggs in Peninsular Malaysia: Implications for Conservation and Public Health

**DOI:** 10.1289/ehp.0900813

**Published:** 2009-05-29

**Authors:** Jason P. van de Merwe, Mary Hodge, Henry A. Olszowy, Joan M. Whittier, Kamarruddin Ibrahim, Shing Y. Lee

**Affiliations:** 1 Griffith School of Environment and Australian Rivers Institute, Griffith University, Gold Coast, Queensland, Australia; 2 Queensland Health Scientific Services, Queensland Government, Coopers Plains, Queensland, Australia; 3 School of Biomedical Sciences, University of Queensland, St Lucia, Queensland, Australia; 4 Turtle and Marine Ecosystems Centre, Department of Fisheries Malaysia, Rantau Abang, Terengganu, Malaysia

**Keywords:** C. mydas, heavy metals, human health, persistent organic pollutants, risk assessments

## Abstract

**Background:**

Persistent organic pollutants (POPs)—such as organochlorine pesticides (OCPs), polychlorinated biphenyls (PCBs), and polybrominated diphenyl ethers (PBDEs)—and heavy metals have been reported in sea turtles at various stages of their life cycle. These chemicals can disrupt development and function of wildlife. Furthermore, in areas such as Peninsular Malaysia, where the human consumption of sea turtle eggs is prevalent, egg contamination may also have public health implications.

**Objective:**

In the present study we investigated conservation and human health risks associated with the chemical contamination of green turtle (*Chelonia mydas*) eggs in Peninsular Malaysia.

**Methods:**

Fifty-five *C. mydas* eggs were collected from markets in Peninsular Malaysia and analyzed for POPs and heavy metals. We conducted screening risk assessments (SRAs) and calculated the percent of acceptable daily intake (ADI) for POPs and metals to assess conservation and human health risks associated with egg contamination.

**Results:**

*C. mydas* eggs were available in 9 of the 33 markets visited. These eggs came from seven nesting areas from as far away as Borneo Malaysia. SRAs indicated a significant risk to embryonic development associated with the observed arsenic concentrations. Furthermore, the concentrations of coplanar PCBs represented 3 300 times the ADI values set by the World Health Organization.

**Conclusions:**

The concentrations of POPs and heavy metals reported in *C. mydas* eggs from markets in Peninsular Malaysia pose considerable risks to sea turtle conservation and human health.

Peninsular Malaysia has historically supported large nesting populations of *Chelonia mydas*, predominantly in the east coast state of Terengganu (Hendrickson and Alfred 1961; Hendrickson and Balasingham 1966). However, *C. mydas* nesting populations in Peninsular Malaysia have declined by > 80% since the 1950s, primarily because of the collection of eggs for human consumption (Hendrickson and Alfred 1961; Ibrahim 1994; Ibrahim et al. 2003; Limpus 1993; Siow and Moll 1981). It has been estimated that prior to the late 1950s, nearly 100% of all sea turtle eggs laid on the beaches of Peninsular Malaysia were collected for human consumption at a rate of up to 2 million eggs per year (Hendrickson and Alfred 1961; Siow and Moll 1981).

Under current legislation, the majority of eggs are protected, with the remaining eggs allocated to collectors under a permit system (Ibrahim et al. 2003). In 2006, it was estimated that 90% of all *C. mydas* eggs laid in Peninsular Malaysia were relocated to hatcheries or protected *in situ* by the Department of Fisheries, Malaysia (Ibrahim K, unpublished data). However, this figure is dependent on Department of Fisheries budgetary allocations and can fluctuate from year to year. Furthermore, considering the high economic value of sea turtle eggs [~ $0.60 1.20 (US$) each], there is an incentive for permit holders to underreport egg collection, and eggs may even be harvested without permits for sale in black markets.

The consumption of sea turtle products (tissues, eggs, and blood) poses a number of public health concerns because of high lipid content and the presence of bacteria, parasites, and environmental contaminants (Aguirre et al. 2006). As a guide for consumption of foods containing environmental contaminants, the World Health Organization (WHO) and other regional organizations have determined acceptable daily intakes (ADIs) of these compounds [Food and Agriculture Organization of the United Nations (FAO)/ WHO 2007]. Persistent organic pollutants (POPs) and heavy metals have been reported in the eggs of a number of *C. mydas* populations (Clark and Krynitsky 1980; Godley et al. 1999; Lam et al. 2006; McKenzie et al. 1999; Podreka et al. 1998; Thompson et al. 1974). Determining the contamination of *C. mydas* eggs in Peninsular Malaysia and estimating the risks associated with consuming these eggs is therefore an important aspect of public health management.

The chemical contamination of *C. mydas* eggs may also have conservation implications. POPs and heavy metals are known to affect development, reproduction, and immune function in fish, birds, reptiles, and mammals (Chang 1996; Colborn et al. 1993; Guillette and Gunderson 2001; McKinlay et al. 2008). Correlative and *in vitro* studies have shown that environmentally relevant concentrations of mercury, polychlorinated biphenyls (PCBs), and 4,4′-DDE (dichloro diphenyl-dichloroethylene) can affect the immune function and health of loggerhead sea turtles, *Caretta caretta* (Day et al. 2007; Keller et al. 2004, 2006a, 2006b). However, the most well-documented effects of POPs in oviparous reptiles are on hatchling development. Eggs of the American alligator (*Alligator mississippiensis*) with high levels of *p,p*′-DDE, *p,p*′-DDD (dichlorodiphenyldichloroethane), dieldrin, and *cis-*chlordane experienced high egg mortality (Guillette and Crain 1996). Similarly, PCBs and organochlorine pesticides (OCPs) in the eggs of the freshwater snapping turtle (*Chelydra serpentina*) have been associated with increased embryonic mortalities and deformities (Bishop et al. 1991; Bryan et al. 1987; de Solla et al. 2008; Helwig and Hora 1983; Olafsson et al. 1983). Similar effects of POPs on the development of sea turtle eggs may therefore be expected.

The chemical contamination of *C. mydas* eggs in Peninsular Malaysia potentially has both conservation and human health consequences. The availability of *C. mydas* eggs in the markets of Peninsular Malaysia provides an opportunity to collect a sample of this turtle population’s eggs and assess the level of chemical contamination. This information can be used to predict the effects of these chemicals on *C. mydas* populations and the potential harm they may cause to the humans consuming them.

## Materials and Methods

### Market egg collection

In August 2006, we conducted a survey by road to identify markets in Peninsular Malaysia selling *C. mydas* eggs for human consumption. Over 6 days, a 1,115-km route covering ~ 730 km of coastline was taken from Kota Bharu through Kuantan, Kuala Lumpur, Johor Bahru, and back up the east coast to Kuantan. Each of the 33 markets encountered on this route was surveyed for *C. mydas* eggs. A random sample of 3 13 eggs was purchased from each market where eggs were being sold. In larger markets with multiple vendors, eggs were taken from a random sample of the vendors. In total, 55 eggs were collected and kept frozen ( 20°C) until analysis. At the time of collection, vendors were asked about the nesting location and the approximate date the eggs had been collected. Although we could not confirm the reliability of these locations and dates, we did not expect the reliability of information to interfere with the interpretation of the calculated risk assessments.

### Chemical analysis

We analyzed egg samples for 83 PCB congeners, 23 OCPs, and 19 polybrominated diphenyl ether (PBDE) congeners using gas chromatography with tandem mass spectrometry (GC-MS/ MS), following previously described methods (van de Merwe et al. 2009). We determined that the limit of detection (LOD) was compound and sample specific, although for most compounds the LOD was < 10 pg/g. The coefficient of variation [SD ÷ (mean × 100)] of 10 replicates of pooled *C. mydas* egg collected from Heron Island (Queensland, Australia) in 1998 was < 20% for all compounds, although generally < 5%. We analyzed avian egg control (QC04-ERM1: common murre, *Uria aalge,* and thick-billed murre, *Uria lomvia*; National Institute of Standards and Technology, Charleston, SC, USA), finding concentrations within 60% of the reference values for all POP compounds (Vander Pol et al. 2007). Recoveries of mass-labeled internal standards ranged from 30 to 96% and were < 60% only for the higher chlorinated PCBs.

We also analyzed eggs for zinc, copper, cobalt, selenium, arsenic, cadmium, and lead using acid digestion [nitric acid (HNO_3_)] and inductively coupled plasma mass spectrometry, following methods modified from Scheelings (2002), Tinggi et al. (2004), and Sakao and Uchida (1999). Eggs were analyzed for mercury using acid digestion (sulfuric acid, HNO_3_, hydrochloric acid) and cold vapor atomic absorption spectrometry, using methods modified from Tinggi and Craven (1996). The limit of reporting was 0.05 μg/g for Cu, Zn, Se, As, and Pb, and 0.01 μg/g for Cd and Hg; the coefficient of variation of 10 replicates of pooled *C. mydas* egg collected from Heron Island in 1998 ranged from 0.5 to 1.9%. We analyzed DORM-I dogfish muscle standard reference material (National Research Council Canada, Ottawa, Ontario, Canada) and Queensland Health Scientific Services (Coopers Plains, Queensland, Australia) in-house seafood mix standard reference materials (QAC 180, QAC 150, and FFM 04), finding values of 88–109% of the certified values for all elements.

### Screening risk assessments (SRAs)

We performed SRAs for a number of the major POPs and metals, using methods previously reported for risk assessments of metals in sea turtle eggs (Lam et al. 2006). Hazard quotients (HQs) were calculated for each compound by dividing the measured concentration (MEC) by the predicted no-effect concentration (PNEC). We generally estimated the PNECs from toxicologic studies on the effects of these compounds on sea turtles and other oviparous reptiles. However, in cases where this information was not available, PNECs were estimated from bird data [see Supplemental Material available online (doi:10.1289/ehp.0900813.S1 via http://dx.doi.org/)]. The HQs were calculated for both a best-case and worst-case scenario (Equations 1 and 2). In cases where there was limited information and a maximum and minimum PNEC could not be determined, the same value was used for both HQs.


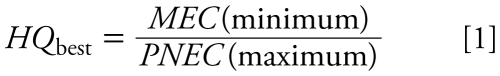



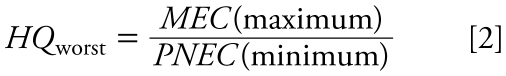


### Human health risk assessments

The FAO/ WHO Joint Expert Committee on Food Additives (JECFA) has calculated ADIs for most POPs and heavy metals (FAO/WHO 2007; WHO 2008). The ADIs are based on human and animal experiments that investigate the no observable adverse effect levels of these chemicals, and are generally presented as micrograms per kilogram of body weight per day. Human health risk assessments generally include the percentage of people within a population with a chemical consumption level above the ADI for each compound (Van Oostdam et al. 2005). However, because the rate of *C. mydas* egg consumption in Peninsular Malaysia was not available and we did not investigate it in the present study, we used the maximum concentration of each of the major POP and metal compounds reported to evaluate risk by expressing the contaminant concentration as percentage of ADI in a single *C. mydas* egg (Equation 3). This calculation was based on a person weighing 65 kg consuming a single *C. mydas* egg of 35 g.


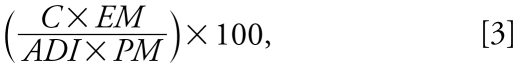


where *C* is the maximum concentration of contaminant reported in *C. mydas* eggs (micrograms per gram), *EM* is the mass of a *C. mydas* egg (35 g), *ADI* is in micrograms per kilogram of body weight per day, and *PM* is a person’s body weight (65 kg).

### Statistical analysis

We arranged POPs into six major groups:

- PCBs: 83 PCB congeners
- OCPs: mirex, endosulfan I, pentachloro-benzene, dieldrin, and heptachlor epoxide
- Chlordanes: oxychlordane, *trans*/*cis*-chlordane, *trans/cis*-nonachlor
- Hexachlorocyclohexanes (HCHs): α-HCH, β-HCH, and-γ-HCH
- Dichlorodiphenyltrichloroethanes (DDTs): 2,4′-DDE, 4,4′-DDE, 2,4′-DDD, 2,4′-DDT, 4,4′-DDD, and 4,4′-DDT
- PBDEs: 19 PBDE congeners.

We calculated the mean concentration (± SE) of each POP group and metal and assigned all values < LOD a value of LOD/2. This produced the least amount of bias while not requiring the use of complex iteration software (Helsel 1990). We also calculated the number of individual POP compounds in each egg.

## Results

### Green turtle eggs in markets of Peninsular Malaysia

*C. mydas* eggs were found in 9 of the 33 markets visited (Table 1). All of these markets were on the east coast of Peninsular Malaysia and ranged from multiple vendors selling hundreds of eggs each (in the larger town markets) to a single vendor selling 50 100 eggs at a roadside fish market. According to the vendors, the eggs had been collected from seven different nesting sites, at times ranging from the previous night to 10 days before purchase for this study (Table 1). The nesting areas from which the eggs had been collected ranged from nearby beaches to as far away as Sabah in Borneo (Malaysia) [see Supplemental Material (doi:10.1289/ehp.0900813.S1)].

We identified OCPs, PCBs, chlordanes, and HCHs in all 55 of the eggs analyzed. Compounds most commonly identified were PCBs 18, 28/31, 52, 56, 74, 99, 118, 132/153, 138/158, 156, 170, 178, 180/193, 183, 187, 194, 196/203, and 202; dieldrin, endosulfan I, heptachlor epoxide, hexachlorobenzene, and mirex; β-HCH and γ-HCH; 4,4′-DDE and 4,4′-DDT; and PBDEs 47, 99, 100, 153, and 154. The OCPs and PCBs were the most highly concentrated POP compounds, whereas chlordanes and HCHs were generally found at trace levels. The PBDEs and DDTs were identified at trace levels in 84% of the eggs analyzed (Table 2). Percent lipids ranged from 7.3% to 13.5% (mean ± SE, 9.3 ± 0.1%).

Of the essential metals, Zn, Cu, and Se were identified in all of the 55 eggs analyzed. However, Co was not detected in any of the eggs. Zn was the most highly concentrated element in each egg. For the toxic metals, As was the most common element with the highest concentrations, detected in 65% of the eggs sampled. Cd and Pb were less common, being detected in 49% and 11% of the eggs, respectively. Hg was not detected in any of the eggs (Table 2).

### Screening risk assessments

The worst-case HQs ranged from < 0.01 to 0.51 for POPs and from 0.1 to 19.5 for heavy metals (Table 3).

### Human health risk assessments

The maximum percent of ADI in one *C. mydas* egg ranged from < 0.1 to 30,247% for POP compounds and from 1.5 to 12.2% for metals (Table 4).

## Discussion

### Availability of green turtle eggs in markets of Peninsular Malaysia

The sale of *C. mydas* eggs was confined to towns on the east coast of Peninsular Malaysia and occurred in only 9 of the 33 markets surveyed. Furthermore, the long-distance transportation of eggs from Sabah and Perak to east coast markets may indicate that the local demand for sea turtle eggs is not currently being met because of the level of egg protection. Between 1996 and 2006, the proportion of *C. mydas* eggs laid in Peninsular Malaysia that were protected in hatcheries or *in situ* by the Department of Fisheries, Malaysia, increased from 13% to 94%. This effectively decreased the number of eggs available for collection and consumption per year from > 170,000 to < 15,000 (Department of Fisheries Malaysia, unpublished data). This drastic decline in supply could cause collection of eggs from other areas to meet the sustained demand.

The importation of *C. mydas* eggs from areas such as Sabah and Perak to compensate for the decline in local supply has ethical and legal implications that sea turtle managers need to consider. Conservation of *C. mydas* in Southeast Asia would be compromised if the protection of eggs in one area increased the collection of eggs in other areas. This would be a particular concern if egg collection increased in nesting habitats that were relatively less disturbed and more difficult to patrol. Furthermore, the transport of *C. mydas* eggs from Sabah to Peninsular Malaysia is illegal under the Convention on the Conservation of Migratory Species of Wild Animals (Convention on Migratory Species 2003) and Sabah state law (Sabah Ministry of Tourism, Culture and Environment 1997). Immediate conservation efforts must therefore increase patrolling and monitoring of egg transport between Borneo and Peninsular Malaysia. However, long-term solutions lie in reducing the demand for—and the consumption of—sea turtle eggs in the region.

The concentrations of POPs and metals in *C. mydas* eggs we found in the present study were generally lower than concentrations previously reported in loggerhead sea turtle (*C . caretta*) eggs (Alam and Brim 2000; Alava et al. 2006; Clark and Krynitsky 1980, 1985; Godley et al. 1999; Sakai et al. 1995; Stoneburner et al. 1980). Additionally, POP and metal concentrations in the present study were considerably different from those reported in previous studies on *C. mydas* eggs (Table 5). Higher concentrations in *C. caretta* eggs may be explained by the higher trophic level occupied by this carnivorous species. Differences in egg contamination between *C. mydas* from different locations may reflect varied regional use of these chemicals. However, the contamination of *C. mydas* is more likely caused by chemical use in the turtles’ foraging areas (Newman and Unger 2003). Therefore, because a *C. mydas* nesting population generally comprises turtles from a wide range of foraging areas (Cheng 2000; Godley et al. 2002; Liew et al. 1995; Seminoff et al. 2008), the precise source of egg contamination is difficult to determine.

### Egg contamination and conservation

The SRAs indicated that the POP concentrations observed in the *C. mydas* eggs collected from markets in Peninsular Malaysia were unlikely to cause sex reversal in the developing embryos. Generally speaking, if the HQ of a particular compound is > 1, that compound is likely to have toxicologic effects (Suter 1993; Tannenbaum et al. 2003). Dieldrin posed the highest risk to sex reversal in the *C. mydas* eggs, although a worst-case HQ of 0.51 indicated that this risk was minimal. However, the metal concentrations observed in these *C. mydas* eggs indicated a higher risk of disruptions to embryonic development. In particular, the concentrations of As (worst-case HQ, 19.5) suggested a relatively high risk of embryonic mortality and reduced hatch success. Similarly, the HQ for Se (2.5) found in these eggs indicated that this metal would likely affect hatching success.

There are many uncertainties in the calculations of PNECs for the SRAs in the present study because of the low number of toxicologic studies specific to *C. mydas* eggs. Furthermore, we calculated the HQs for the toxicologic effects of single compounds acting alone. The present study is the first of its kind to analyze and identify such a large number of POP and metal compounds in *C. mydas* eggs. The large number of POP compounds we observed in the eggs (mean ± SE, 27.0 ± 0.8; range, 17 45) could therefore be important in terms of combined effects. As previously reported in *Trachemys scripta*, the combination of a trichlorinated biphenyl with a tetra-chlorinated biphenyl produced sex reversal at a lower concentration than when the PCBs were administered individually (Bergeron et al. 1994). The effect of multiple PCB compounds on sex reversal in the present study may therefore be more important than the HQs have indicated.

### Egg contamination and human health

The most alarming statistic from the assessment of the human health risk associated with consuming *C. mydas* eggs in Peninsular Malaysia was related to coplanar PCBs.

The human health risk assessment we calculated in the present study indicated that the consumption of a single *C. mydas* egg can result in the intake of > 300 times the ADI of coplanar PCBs, as determined by JECFA (FAO/WHO 2007). Even though this represents the maximum risk calculated, coplanar PCBs were detected in all 55 *C. mydas* eggs analyzed. Even the consumption of an egg with the lowest concentration of coplanar PCBs reported would result in the intake of more than 3 times the ADI. Coplanar PCBs are particularly toxic and are often considered in the same context as dioxins and furans, some of the most toxic chemicals known (McKinlay et al. 2008). The reported concentrations of coplanar PCBs therefore create serious health risks to humans who consume *C. mydas* eggs in Peninsular Malaysia.

The risks associated with consuming *C. mydas* eggs in the present study are also likely to be an underestimation of the overall risk of these chemicals to human health in this region. Other foods consumed by the people of Peninsular Malaysia may also be contaminated with POPs and heavy metals. If these chemicals were also being consumed through other dietary intake, the contribution from *C. mydas* eggs would become more significant. In a recent global study on the contamination of skipjack tuna (*Katsuwonus pelamis*), concentrations of coplanar PCBs were highest in Southeast Asia (Ueno et al. 2005). Furthermore, high levels of other contaminants have been reported in the seafood of this region (Agusa et al. 2007). The human health risks associated with consuming contaminated food is therefore of particular concern in coastal communities of Southeast Asia. In addition, the ADIs are generally calculated on an individual compound basis (FAO/ WHO 2007). The effects of consuming foods containing multiple chemical compounds are not well understood. Despite the fact that the concentrations of coplanar PCBs indicate a high risk, the large number of chemicals found in *C. mydas* eggs may further increase the human health risks associated with consuming these eggs.

Incidentally, the chemical contamination found in the *C. mydas* eggs in Peninsular Malaysia may assist conservation efforts in the region. The health risk of consuming eggs contaminated with various chemical compounds could potentially reduce the number of eggs collected for consumption, which in turn would allow an increase in sea turtle populations in Southeast Asia. Although there are alternative, cost-effective sources of protein available to east coast communities of Peninsular Malaysia (e.g., fish, chicken, beef), it is the cultural perception that sea turtle eggs have medicinal qualities that primarily drives their consumption. One of the greatest challenges to sea turtle conservation agencies in this region is to reduce this cultural demand for sea turtle eggs. This could be achieved by awareness campaigns highlighting the health consequences of consuming sea turtle eggs containing such a large number of toxic compounds. If effective, this would presumably reduce the collection of sea turtle eggs for human consumption and contribute significantly to sea turtle conservation efforts in the region.

## Figures and Tables

**Table 1:** The nesting locations and time between collection and purchase of *C. mydas* eggs from markets in Peninsular Malaysia.

Market	Egg collection location	Distance from market (km)	Days since collection
Kota Bharu	Turtle Island (Borneo)	1,800	10
	Redang/Perhentian Islands	80	7
Pasir Putih	Tioman Island	400	8
Penarik	Punti Bari	2	1
Kuala Terengganu	Redang Island	65	1
	Turtle Island (Borneo)	1,700	3
	Perak	300	2
Paka	Paka beach	1	1
Dungun	Paka beach	1	3–7
Mersing	Tioman Island	55	1–7
Cukai	Cherating	10	7–10
Kuantan	Turtle Island (Borneo)	1,700	3–6

**Table 2:** Concentrations of POPs (pg/g wet mass) and metals (μg/g wet mass) detected in *C. mydas* eggs (*n* = 55) from the markets of Peninsular Malaysia.

Compound	Mean ± SE	Range (*n*)^a^
POPs		
∑PCBs	470.5 ± 83.3	146.6–3691.5
∑OCPs	394.9 ± 43.1	169.5–2286.7
∑Chlordanes	57.5 ± 9.4	24.7–514.2
∑HCHs	68.8 ± 8.7	13.2–230.1
∑DDTs	83.5 ± 18.3	LOD–701.9 (46)
∑PBDEs	21.4 ± 6.6	LOD–352.7 (46)
∑POPs	1096.6 ± 432.5	432.5– 6266.9
No. of POPs	27.0 ± 0.8	17–45
Essential metals		
Copper	0.526 ± 0.023	0.056–1.073
Zinc	15.34 ± 0.93	1.33–39.48
Selenium	0.464 ± 0.026	0.049–0.836
∑Essential	16.33 ± 0.96	1.44–40.92
Toxic metals		
Arsenic	0.097 ± 0.011	LOD–0.351 (36)
Cadmium	0.009 ± 0.001	LOD–0.029 (27)
Lead	0.031 ± 0.003	LOD–0.124 (6)
∑Toxic	0.138 ± 0.012	LOD–0.380 (46)
Lipids (%)	9.33 ± 0.14	7.3–13.54

∑ sum of compounds.

a Number of samples > LOD is shown in parentheses.

**Table 3:** The best and worst case HQs for metals and POPs identified in *C. mydas* eggs from markets in Peninsular Malaysia.

Compound	MEC_min_	MEC_max_	PNEC_min_	PNEC_max_	HQ_worst_	HQ_best_
POPs (ng/g)						
DDTs^a^	0	0.702	543	543	< 0.01	0
Dieldrin	0.008	2.02	4	4	0.51	< 0.01
Tri-CBs^b^	0	0.512	26	26	< 0.01	0
Tetra-CBs^c^	0.060	2.00	26	26	< 0.01	< 0.01
∑ PCBs^d^	0.147	3.69	26	26	0.14	< 0.01
Metals (μg/g)						
Pb	0	0.124	1	1	0.1	0
Se	0.049	0.836	0.34	6	2.5	< 0.01
Cd	0	0.029	0.0014	0.013	0.2	0
As	0	0.351	0.018	0.018	19.5	0

Abbreviations, ∑, sum of compounds; max, maximum; min, minimum.

a The sum of DDTs was used for MECs, but values for PNEC were derived from effects of *p,p′*-DDE.

b The sum of tri-chlorinated biphenyls was used for MECs, but values for PNEC were derived from effects of a single trichlorinated biphenyl.

c The sum of tetrachlorinated biphenyls was used for the MECs, but values for PNEC were derived from effects of a single tetrachlorinated biphenyl.

d The sum of all PCBs was used for the MECs but values for PNEC were derived from effects of a single tetrachlorinated biphenyl.

**Table 4:** The maximum percentage of ADI in one *C. mydas* egg for the major POPs and metal compounds reported in eggs from Peninsular Malaysia.

Compound	ADI^a^ (μg/kg/day)	Egg concentration (ng/g wet mass)	Maximum percent of ADI in one egg^b^
POPs			
PBDEs	100	< LOD–0.353	< 0.1
DDT	20	< LOD–0.702	< 0.1
Endosulfan	6	0.104–0.271	< 0.1
PCBs	1	0.147–3.69	0.2
HCH	0.3	0.013–0.231	< 0.1
Dieldrin	0.1	0.008–2.02	1.1
Mirex	0.07	< LOD–0.027	< 0.1
Chlordane	0.05	0.025–0.514	0.6
Coplanar PCBs	2 × 10^−6^	0.014–1.29	30,247
Metals			
Zn	300–1,000	1,330–39,480	9.1^c^
Cu	50–500	56–1,070	1.5^c^
Se	12.5	49–836	4.6
Pb	3.6	< LOD–124	2.4
As	2	< LOD–351	12.2
Cd	1	< LOD–29	2.0

a ADI determined by the JECFA and the International Programme on Chemical Safety (FAO/WHO 2007; WHO 2008).

b Calculated based on a person weighing 65 kg consuming a single 35-g *C. mydas* egg.

c Calculated from the most conservative ADI and maximum concentration of zinc reported (FAO/WHO 2007).

**Table 5:** Summary of the concentrations (mean ± SE or range) of common POP compounds (ng/g wet mass) and heavy metals (μg/g wet mass) reported in *C. mydas* eggs from previous studies.

Location	No. of eggs	*p,p*′-DDE	∑ PCBs	Cd	Pb	Reference
Florida (USA)	2	2.0 ± 2.0	—	—	—	Clark and Krynitsky 1980
Ascension Island^a^	10	< LOD–9	20–220	—	—	Thompson et al. 1974
Heron Island^b^	15	1.7 ± 0.3	—	—	—	Podreka et al. 1998
Hong Kong	30	—	—	< LOD	0.03–0.14	Lam et al. 2006
Cyprus	17	—	—	0.05–1.2	LOD–1.61	Godley et al. 1999
Peninsular Malaysia	55	< LOD	0.15–3.7	LOD–0.03	LOD–0.12	Present study

—, Not investigated.

a Island in the South Atlantic Ocean.

b Queensland, Australia.
